# Visual Perceptual Difficulties and Under-Achievement at School in a Large Community-Based Sample of Children

**DOI:** 10.1371/journal.pone.0014772

**Published:** 2011-03-21

**Authors:** Cathy Williams, Kate Northstone, Ricardo Sabates, Leon Feinstein, Alan Emond, Gordon N. Dutton

**Affiliations:** 1 School of Social and Community Medicine, University of Bristol, Bristol, United Kingdom; 2 Department of Education, University of Sussex, Brighton, United Kingdom; 3 Institute of Education, University of London, London, United Kingdom; 4 Royal Hospital for Sick Children and Department of Visual Sciences, Glasgow Caledonian University, Glasgow, United Kingdom; University of Sydney, Australia

## Abstract

**Introduction:**

Difficulties with visual perception (VP) are often described in children with neurological or developmental problems. However, there are few data regarding the range of visual perceptual abilities in populations of normal children, or on the impact of these abilities on children's day-to-day functioning.

**Methods:**

Data were obtained for 4512 participants in an ongoing birth cohort study (Avon Longitudinal Study of Parents and Children; ALSPAC). The children's mothers responded to questions designed to elicit indications of visual perceptual difficulties or immaturity, when their children were aged 13 years. We examined associations with standardised school test results in reading and in mathematics at age 13–14 years (SATS-KS3), accounting for potential confounders including IQ.

**Results:**

Three underlying factors explained half the variance in the VP question responses. These correlated best with questions on interpreting cluttered scenes; guidance of movement and face recognition. The adjusted parameter estimates (95% CI) for the cluttered-scenes factor (0.05; 0.02 to 0.08; p<0.001) suggested positive associations with the reading test results whilst that for the guidance-of-movement factor (0.03; 0.00 to 0.06; p = 0.026) suggested positive association with the mathematics results. The raw scores were associated with both test results.

**Discussion:**

VP abilities were widely distributed in this sample of 13-year old children. Lower levels of VP function were associated with under-achievement in reading and in mathematics. Simple interventions can help children with VP difficulties, so research is needed into practicable, cost-effective strategies for identification and assessment, so that support can be targeted appropriately.

## Introduction

Neuronal injury is now the commonest cause of blindness or severe visual impairment amongst children in the UK [1]. Brain injury or malfunction is also associated with a range of less severe, but functionally important visual difficulties including visual field defects, eye movement disorders and difficulties in image processing or interpretation, which are also known as visual perceptual (or visual cognitive) problems. Although not routinely tested for in most paediatric eye clinics, visual perceptual (VP) problems are well recognized sequelae of many conditions including periventricular leucomalacia[2], [3], cerebral palsy [4] and hydrocephalus[5]. VP abilities improve during infancy and childhood in normal development [6], [7], [8], [9], [10] but development may be delayed or impaired in neurogenetic disorders such as Williams syndrome [11], [12], [13], [14]. Deficits in visual perceptual abilities may coexist with other neurodevelopmental problems such as reduced performance in intelligence tests [15], or may be isolated and unassociated with other cognitive deficits [16].

There are many VP functions or abilities described, with various neural substrates thought to be responsible for them. Examples include visual attention (the ability to highlight specific features or places within the visual field); visual search (the ability to move the eyes within a scene to detect relevant targets); perceptual grouping (the ability to combine components of a scene into a meaningful whole); unconscious use of visuospatial information to programme movements that interact with objects in 3-dimensional space; route-finding and recognition of objects and people. Although there are many hypotheses relating to the neural mechanisms producing these and other abilities, a widely-used current model is that there are two important networks for visual information; the “dorsal stream” which links the occipital lobe with the parietal lobe and is preferentially active for immediately and subconsciously judging “where” an object is and how to reach or interact with it, and the “ventral stream” which links the occipital and temporal lobes and is preferentially active for judgements about “what” something looks like (for example recognition of faces, objects)[17]. Whilst these networks work together much of the time, they can be activated to different degrees by different types of visually-demanding tasks, thus supporting the hypothesis of anatomically distinct areas of functional specialisation within the visual system [18], [19], [20].

A few studies have reported that children's visual perceptual abilities can appear differently in real-world as compared with experimental tasks [21]. Children at risk of visual perceptual problems, such as those born very prematurely, are known to have increased rates of behavioural problems [22] and educational difficulties requiring support [23] but whether visual perceptual problems contribute to these outcomes is not known. Similarly there is little information about individual variability in visual perceptual skills amongst children in the general population, to provide a context for the visual perceptual deficits reported in groups of patients.

We used data from an ongoing geographically-based birth cohort study to estimate the prevalence in healthy children of anomalies of visual behaviour that in clinical subjects would suggest visual perceptual problems. We examined the associations between those symptoms and the children's educational outcomes. We hypothesized that maternally-reported behaviour suggestive of poor visual perceptual abilities would be associated with difficulties in learning and result in reduced educational attainment.

## Results

School outcome data (reading or maths KS3 results) and complete maternal responses to the VP questions were available for 4512 children. The characteristics of the children included in this analysis are shown in Table 1, as are the characteristics of the imputed data and of the ALSPAC children who were excluded. Children included in this analysis were less likely to be from very advantaged or very disadvantaged families; less likely to have a mother with only the lowest level of education, or with a degree; less likely to have an ICD-10 diagnosis affecting development and less likely to have been born preterm. Overall, the KS3 results were slightly higher for the children we included compared to those who were excluded. The imputed data we generated are broadly similar to the data of the excluded children, but with lower mean KS3 results, lower IQ scores, fewer degree-level mothers and more disadvantaged families.

**Table 1:** Factor loadings (pearson correlation coefficients) between PCA-derived Visual Perception factors and the individual questions asked.

	Factor 1	Factor 2	Factor 3
Recognises members of close family	0.008	0.016	0.806
Recognises friends	0.109	0.082	0.798
Recognises people from photographs	0.498	−0.052	0.388
Loses objects around house	0.357	0.304	0.031
Difficulty grasping objects	0.017	0.770	0.047
Difficulty distinguishing step from line	0.029	0.761	0.024
Find objects on patterned carpet	0.689	0.125	0.056
Find objects in complex pictures	0.780	0.029	0.045
Misjudges doorways/corridors	0.154	0.481	0.007
Finds way around house	0.047	0.045	0.015
Difficulty seeing things in distance	0.435	0.209	0.019
Find way in new surroundings	0.649	0.014	0.021

**Figure 1:**
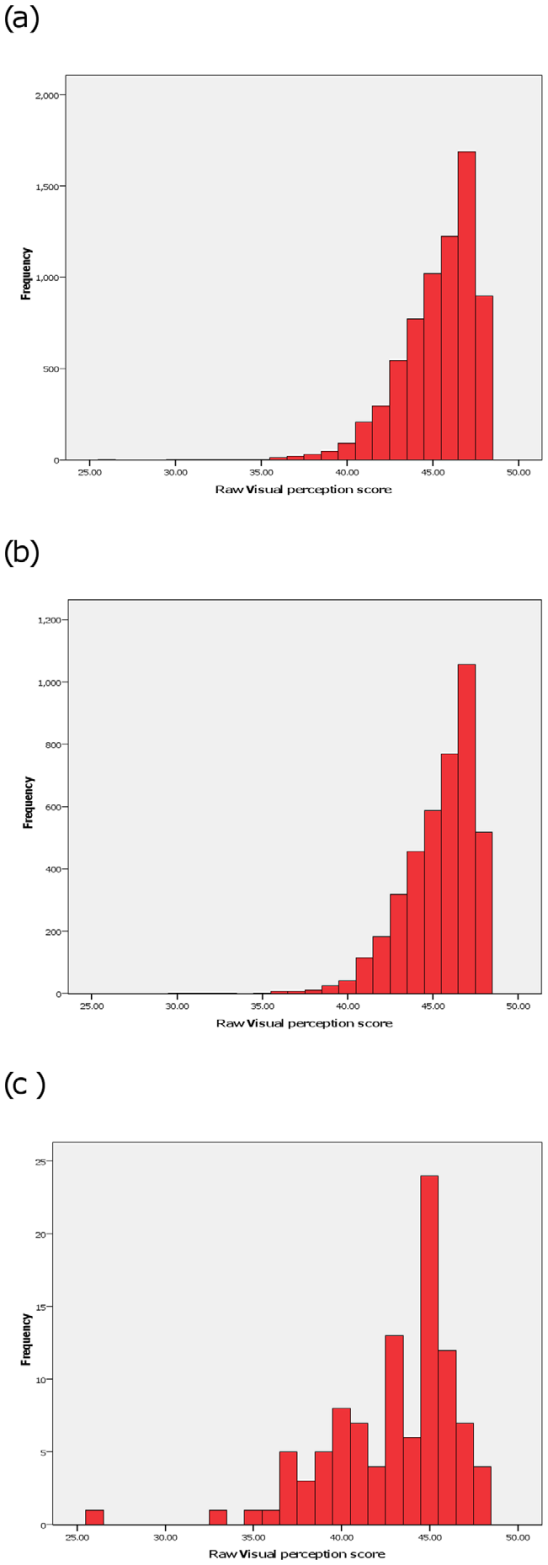
Distributions of raw scores obtained by summing maternal responses to 12 questions on visuoperceptual abilities in their 13-year old children. Legend:(a) all who responded (n = 6870), (b) included in analysis (4414) and (c) with an ICD-10 diagnosis affecting development (n = 102).

The mean reading score at KS3 was 4.59 (SD 1.32) and that for mathematics was 4.69 (SD 1.15). The distribution of the raw scores from the VP questions (Fig. 1) was unimodal, and negatively skewed with a tail representing children with low scores (corresponding to more difficulty in the scenarios the questions asked about). The patterns of distribution were similar for all those whose mothers answered the question (Fig. 1a), those included in this analysis (Fig. 1b) and children in the analysis who had ICD10 diagnoses that might affect development (Fig. 1c). The mean raw scores for these samples (all, included, ICD-10) were 45.3, 45.4 and 42.9 respectively.

The principal components analysis (PCA) of these questionnaire data produced three factors. The factor loadings, which show the extent to which each question is correlated with each factor are shown in Table 2. The first point to note is that each factor had at least some correlation with the majority of questions although the strength of the correlations varied somewhat between factors. The highest two factor loadings for each factor (ie had the highest correlation coefficient) were “finding objects in a complex picture” and “finding things on a patterned carpet” for factor 1; “difficulty grasping objects” and “difficulty distinguishing a step from a line on the ground” for factor 2 and “difficulty recognising friends” or “difficulty recognising family” for factor 3. Based on these results we interpret factor 1 as representing one or more aspects of visual functioning that are particularly necessary for acquiring information from a cluttered visual scene, which we have summarised as “crowded scenes”; and factor 2 as being especially related to the ability to use visual information to guide making accurate movements. Factor 3 is more specifically associated with the questions on facial recognition. The remaining 6 questions had less strong and less specific associations with factors 1–3, suggesting they ask about tasks that need a combination of VP traits, although all contribute to the factor scores that were obtained for each child (the sum of the question responses multiplied by the respective factor loadings).

**Table 2:** Comparison of the observed and imputed data for those with Visual perception scores and KS3 Maths and English scores (n = 4512) and for children excluded from the analysis.

	Observedn	%	Imputedn	%	Excludedn	%	P-value*
**Maternal education**	**4392**		**120**		**8183**		**<0.0001**
CSE or less		14.9		21.9		23.1	
Vocational		9.6		11.0		10.0	
O level		39.3		38.0		32.1	
A level		24.5		20.3		21.3	
Degree		11.7		8.8		13.4	
**Social class**	**4200**		**312**		**7455**		**<0.0001**
I		11.5		7.8		14.2	
II		43.9		35.5		40.4	
III NM		28.4		29.3		23.9	
III M		11.5		17.7		14.7	
IV		4.1		8.4		5.6	
V		0.5		1.3		1.1	
**ICD**	**4512**		**0**		**10811**		**<0.0001**
No		98.6				97.2	
Yes		1.4				2.8	
**Visual problems**	**3356**		**1156**		**3939**		**0.023**
No		97.9		96.1		97.1	
Yes		2.1		3.9		2.9	
**Pre term**	**4512**		**0**		**10219**		**<0.0001**
No		94.6				88.1	
Yes		5.4				11.9	
**SCBU**	**4368**		**44**		**7954**		**0.108**
No		93.3		91.4		92.7	
Yes		6.7		8.6		7.3	
**IUGR**	**4375**		**137**		**9258**		**0.302**
No		90.4		85.9		89.8	
Yes		9.6		14.1		10.2	
**IQ: mean**	**3607**	**103.99**	**905**	**98.7**	**3811**	**104.3**	**0.085**
**KS2 maths: mean**	**4442**	**4.69**	**70**	**3.26**	**5757**	**4.34**	**<0.0001**
**KS2 reading: mean**	**4271**	**4.59**	**241**	**2.70**	**5559**	**4.25**	**<0.0001**

*Comparing those included (observed) to those excluded.

Table 3 shows unadjusted and adjusted parameter estimates from regression models with KS3 reading or mathematics results as the outcome and either the raw scores from the VP questions, or the derived VP factors 1–3, as predictors. There are associations between higher raw scores and higher “crowded scene” scores with higher reading results and a trend towards an association between higher guidance-of-movement scores and better reading results. These are attenuated after the multiple adjustments in Model 1 but there is still evidence to suggest that on average, better overall raw scores or “crowded scene” scores are associated with better reading test results.

**Table 3:** Parameter estimates (β) for associations between VP abilities and school test results at 13–14 years in ALSPAC participants.

OUTCOME	VP ABILITIES	CASES WITH COMPLETE DATA					
		Unadjusted (n = 4512)		Model 1 (n = 2968)		Model 2 (n = 2724)	
		β (95% CI)	p	β (95% CI)	p	β (95% CI)	p
**Reading**	**All Questions**	0.04 (0.03, 0.06)	*<0.0001*	0.03 (0.01, 0.04)	*<0.0001*	0.01 (0.00, 0.02)	*0.025*
	**Factor 1**	0.10 (0.07, 0.13)	*<0.0001*	0.05 (0.02, 0.08)	*0.001*	0.02 (−0.01, 0.05)	*0074*
	**Factor 2**	0.03 (−0.01, 0.06)	*0.063*	0.03 (0.00, 0.05)	*0.061*	0.02 (−0.01, 0.04)	*0.178*
	**Factor 3**	0.01 (0.01,0.05)	*0.204*	0.01 (−0.03, 0.04)	*0.725*	−0.01 (−0.04, 0.01)	*0.286*
**Mathematics**	**All Questions**	0.07 (0.05, 0.09)	*<0.0001*	0.02 (0.00, 0.03)	*0.016*	0.00 (−0.02, 0.01)	*0.395*
	**Factor 1**	0.14 (0.11, 0.18)	*<0.0001*	0.02 (−0.01, 0.06)	*0.150*	−0.01 (−0.03, 0.01)	*0.435*
	**Factor 2**	0.06 (0.03, 0.10)	*<0.0001*	0.03 (0.00, 0.06)	*0.026*	0.00 (−0.02, 0.02)	*0.974*
	**Factor 3**	0.01 (−0.03, 0.04)	*0.759*	−0.001 (−0.04, 0.03)	*0.644*	−0.01 (−0.03, 0.01)	*0.491*

				ALL CASES – IMPUTED DATA (n = 4512)			
				β (95% CI)	p	β (95% CI)	p
**Reading**	**All Questions**			0.02 (0.01, 0.03)	*<0.0001*	0.01 (−0.01, 0.02)	*0.218*
	**Factor 1**			0.04 (0.02, 0.07)	*0.002*	0.01 (−0.01, 0.03)	*0.523*
	**Factor 2**			0.03 (0.01, 0.05)	*0.042*	0.01 (−0.01, 0.04)	*0.197*
	**Factor 3**			−0.05 (0.03, 0.02)	*0.918*	0.00 (−0.02, 0.02)	*0.812*
**Mathematics**	**All Questions**			0.02 (0.01, 0.03)	*0.001*	0.00 (−0.01, 0.01)	*0.348*
	**Factor 1**			0.03 (0.01, 0.06)	*0.012*	−0.01 (−0.03, 0.01)	*0.356*
	**Factor 2**			0.03 (0.01, 0.06)	*0.015*	0.00 (−0.02, 0.02)	*0.985*
	**Factor 3**			0.01 (−0.04, 0.01)	*0.354*	−0.01 (−0.03, 0.01)	*0.125*

Legend for Table 3.

“All questions” refers to the score obtained by summing for each child all responses to questions about visual perceptual (VP) abilities.

*Model 1 is adjusted for Age at KS3 testing; Gender; Maternal education; Highest maternal/paternal social class; ICD10 diagnosis; visual problems, born at less than 37 weeks gestation; admitted to a Special Care Baby Unit in first month; low birthweight; total IQ.

**Model 2 is model 1 and additional adjustment for KS2 results.

The parameter estimates are reduced but still show a similar direction after adjusting for earlier performance in the KS2 exams (at 11–12 years), suggesting the associations may be stronger in the KS3 test results than it was in the KS2 test results. There is no evidence to suggest an association between factor 3 (face recognition) scores and reading results.

By contrast the data suggest that better mathematics results were associated with higher guidance-of-movement (factor 2) scores, rather than “crowded scene” scores, although again the raw scores were also predictive. For the mathematics results there was no suggestion of any association after adjusting for KS2 results.

The results from the imputed dataset (Table 3) are broadly similar to the complete case analyses, although they suggest stronger associations between the raw, “crowded scene” and guidance-of-movement scores with the mathematics results, compared to those evident in the complete case analyses. The imputed results for reading were very similar to the complete case analyses.

**Figure 2:**
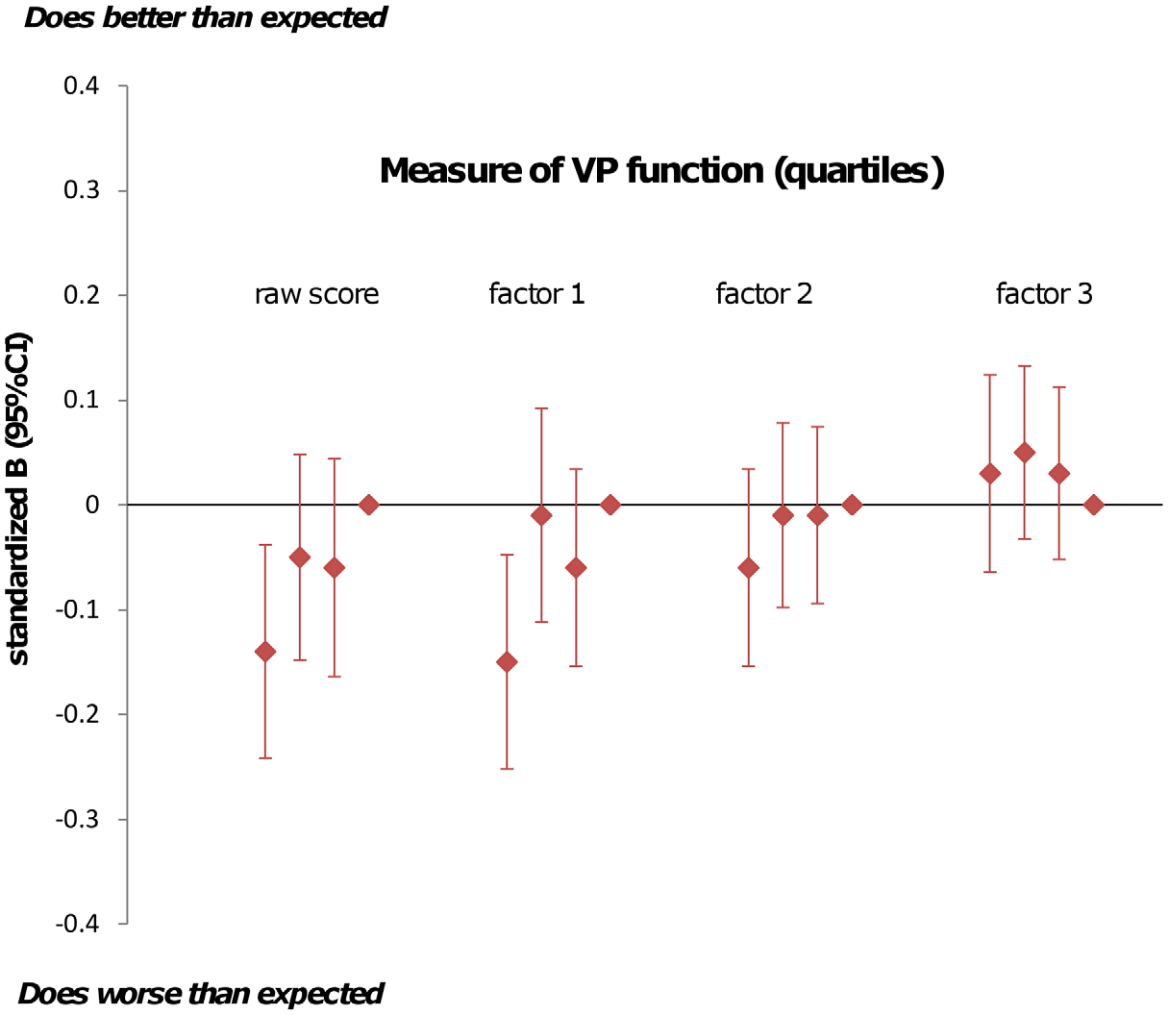
Parameter estimates for measures of visual perception (in quartiles) as predictors of school reading test results. Legend:Analyses adjusted for age at KS3 testing; Gender; Maternal education; Highest maternal/paternal social class; ICD10 diagnosis; visual problems, born at less than 37 weeks gestation; admitted to a Special Care Baby Unit in first month; low birthweight; IQ.

**Figure 3:**
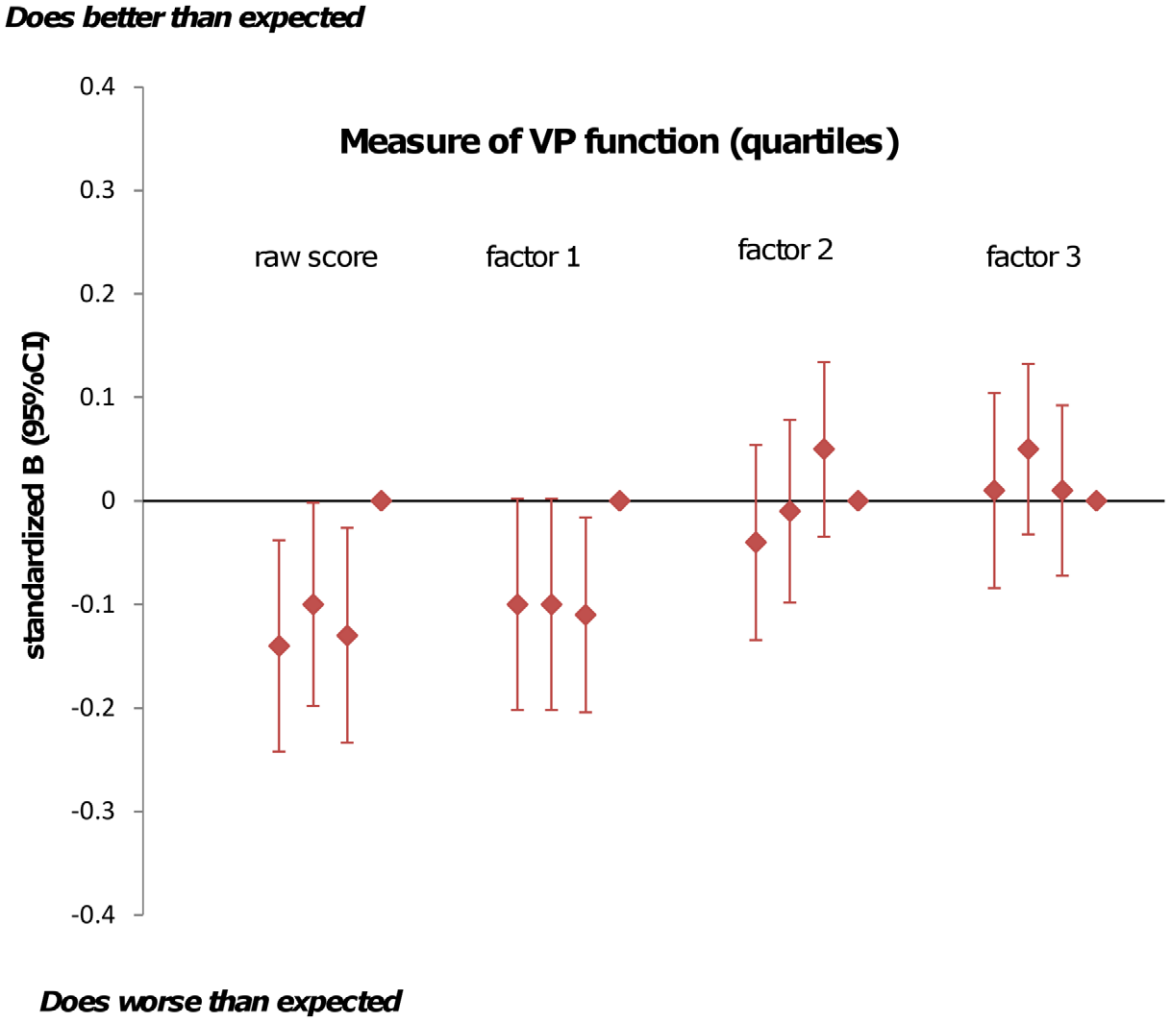
Parameter estimates for measures of visual perception (in quartiles) as predictors of school mathematics test results. Analyses adjusted for age at KS3 testing; Gender; Maternal education; Highest maternal/paternal social class; ICD10 diagnosis; visual problems, born at less than 37 weeks gestation; admitted to a Special Care Baby Unit in first month; low birthweight; IQ.

Figure 2 shows the parameter estimates for reading test results by quartiles of VP performance where the reference category is the top quartile (best VP performance). The association with reading scores is mainly seen for the worst-scoring quartile of raw or “crowded scene” scores. Figure 3 shows comparable data for the mathematics test results and illustrates that the high scoring children for raw, “crowded scene” and guidance-of-movement scores tend to do better in their mathematics tests than the other children, but there is marked variation.

## Discussion

In this community-based sample of 13-year old children we observed a wide spread in the results from maternal reports of their child's visual perceptual abilities. The distribution of our results may suggest that these abilities mature at different rates in children, that there is variation between individuals at similar levels of maturity and/or that there exist in the sample children with undiagnosed neurocognitive problems. The distribution in children with known developmental problems was similarly broad but shifted to the left (towards lower levels of ability). These data suggest that, at this age, there is considerable variability both within clinical groups and amongst healthy children with no apparent developmental problems. This observation is similar to a report of variability within age groups for 385 “super-normal” children aged 6–18 performing a range of cognitive tasks[24]. The study also reported clear increases in ability with age for all cognitive tests studied between 6 – 18 years, with the greatest increases before approximately 12 years of age.

The PCA analysis of the raw questionnaire responses suggested three factors underlying the responses. These factors fit moderately well with the VP functions or traits for which the questions were designed: factor 1 relates most strongly to the ability to see target objects within a crowded scene and is therefore indicative of visual attention and related skills; factor 2 relates to visual guidance of movement and factor 3 to face recognition as an example of ventral stream function. Visual attention and visuospatial/visuomotor skills are thought to be mediated to a considerable extent by the dorsal stream and profound difficulties with these functions are well described in Balint's syndrome, a triad of VP problems associated with bilateral parietal lobe damage; *simultanagnosia* (inability to see objects in a crowded scene despite being able to see them when presented in isolation), *optic ataxia* (inability to accurately reach for objects in the visual field) and optic *apraxia* (inability to make voluntary saccades away from an object of regard despite intact eye movements) [25], [26], [27]. Balint's syndrome has been described in children [28], [29] and has been associated with difficulties in some aspects of reading [30]. Our observation that children with lower scores in factor 1 (“crowded scenes”) do disproportionately badly in their SATS reading test suggests that within the population, relative or absolute deficiencies in the visual attributes represented by this factor may be a real disadvantage in the classroom. The effect size was reduced, as would be expected, after adjustment for other important predictors known to affect school performance such as IQ and socioeconomic background, but remained robust so that children with “crowded scene” scores in the lowest 20% of the sample under-achieved by on average 0.14 SAT levels (see figure 2), equivalent to approximately 3–4 months of predicted progress in their reading test. Thus the effect size attributable to VP difficulties alone is modest but could be an important contributor to underachievement in children with other risk factors for educational disadvantage.

The data presented here support other studies which indicate that dorsal stream function is important for reading and recent reviews have highlighted this body of evidence[31]. However there is also evidence that the ventral stream is important for word recognition [32] and that normal reading relies on many brain areas, including both visual streams [33]. The lack of association between the “ventral” questions we asked and the reading scores, despite much evidence that the ventral stream is important for reading, may be because we asked exclusively about face recognition and we did not ask about other types of object or word recognition. However, the aim of our study was to estimate the impact, if any, of variations in VP abilities on academic achievement within this population of older school children, rather than to investigate the neural substrates involved in reading.

There are several limitations of this study. Firstly, we used a prototype question battery not designed for screening normal populations, but subsequent work with this question inventory is ongoing to develop it as a validated tool for use in normal and clinical populations (G Dutton - in preparation). Secondly, the assessment of VP functions was indirect, as it was reported by the mothers and was not based on the account of the children themselves or by direct observation. The children were relatively old by the time the mothers were asked to report on their childrens' abilities - both the clinical and developmental literature would suggest that VP problems might have been more prevalent and/or more marked when the children were younger and less likely to have developed adaptive strategies. Thus our analyses might have underestimated the strength of association seen between VP abilities and school performance in younger children. Thirdly, our interpretation of what each factor might represent is descriptive, as tasks involving vision rely on the integrity of many different aspects of visual function and specific experimental paradigms are required to illustrate these different aspects in isolation. We aimed to provide the reader with an approximation for the attributes we consider to be related to each of the derived factors, rather than to imply that the derived factors represent mutually exclusive aspects of visual function. Thus our results should not be over-interpreted with respect to the exact VP functions involved in reading or mathematics.

Our study uses some data (IQ, vision defects) that were already available within ALSPAC rather than collecting all relevant data *de novo.* However the main predictor and outcome data were both collected when the children were aged 13–14 years and evidence suggests IQ is relatively stable across childhood [34], and that most childhood eye defects are evident by age 7 [35]. Therefore it is unlikely that important changes in either IQ or the presence of visual defects will have occurred between when these data were collected at 7–8 years and the time of the main data collection at 13–14 years. Children with developmental impairments affecting their education were identified from NHS and education records rather than by a structured research assessment, therefore children with mild developmental delay may not have been identified. Finally, there is some bias in our sample of the ALSPAC cohort as children from very advantaged and very disadvantaged backgrounds are under-represented. As with all observational studies, some confounding may remain despite the statistical adjustments made.

The strengths of our study include the large sample size and the prospective data collection. We have observed robust associations that support hypotheses based on experimental and clinical data, within a community, population-based sample. We have been able to include several important confounders and have imputed the missing data, with largely similar results. Specifically we have taken account of ocular problems such as strabismus, reduced visual acuity and reduced stereopsis, and of any past history that would put a child at risk of more severe visual defects such as field defects, and we found our results were not explained by these, supporting the hypothesis that it is the VP abilities that are responsible for our observed associations, rather than any other visual defects.

These data are important because they suggest a possible cause of academic underachievement for some children. More research is now needed into practicable and cost-effective methods to identify VP difficulties, into the appropriate level of difficulty warranting intervention and into the effectiveness of interventions, all within the context of the existing provisions for medical and educational support for school children and the current financial constraints. However, as simple strategies already exist to help children with VP difficulties [36], this potential cause of academic underachievement could be amenable to intervention (whilst many others are not) - therefore the implications of these data suggest potential ways forward to improve outcomes for children under-achieving at school.

## Methods

### Participants

We used data available from the ongoing Avon Longitudinal Study of Parents and Children (ALSPAC). The inclusion criteria for ALSPAC were to be resident in Avon (an area in the southwest of the UK) and to have an estimated date of delivery between 1/4/91 and 31/12/92; 14451 women were recruited during pregnancy and 13988 children were alive and participating at age 1 year [37]. Comparison with data from the 1991 UK census suggests that the ALSPAC sample was broadly representative of the UK population at the time, with an under-representation of very disadvantaged families and very young mothers [38]. Data collection from the children and their families has been by various methods including self-completion questionnaires sent to the mother, to her partner and after age 5 to the child; direct assessments and interviews in a research clinic; biological samples and linkage to school and hospital records.

### Ethics and consent

Detailed written information about the study was provided at enrolment. Informed consent was obtained in writing for all examinations of the child and was implicit on receipt of completed questionnaires from the mother. This study was approved by the ALSPAC Law and Ethics committee and by all relevant local research ethics committees.

### Outcome data

The results of the school-administered Standardised Attainment Test Scores (SATS) at age 10–11 years (“key stage 2”, KS2) and at 13–14 years (“key stage 3”, KS3) were obtained from the UK government Department of Children, Schools and Families. SATS tests are scored in each subject at levels 1–8. UK Government recommendations are that children should achieve at least a level “4” by key Stage 2 (10–11 years), and a level “5” by Key Stage 3 (13–14 years) [39] and that they should progress by at least by 1.0 unit or level every two years. Thus a coefficient of 0.5 represents an average one year of progress. We used the results for reading and for mathematics and these were each recalibrated to form a continuous score (representing fractions of a standard level or unit) according to the method of Levacic et al [40]. This method adjusts for the different levels of difficulty in the specific test papers children were given, which were chosen according to their anticipated abilities.

### Predictor data

Mothers taking part in the ALSPAC study have been sent questionnaires at regular intervals since their child was born, each asking a range of questions about the child's current activities and development. When the children were aged 13, the questionnaire sent to the mothers included a specially adapted set of 12 questions, which had been used previously in clinical settings to identify children with VP difficulties [41]. The individual questions are listed in Table 1 and for each one, the mother was asked whether the child currently had difficulties (or could manage easily) in specific situations. The options were: “always”, “often”, “sometimes”, or “never”. Three of the questions relate to face recognition (intended as questions about ventral stream capabilities) and 9 questions relate to activities more reliant on dorsal stream activities: route-finding, subconscious visual guidance of movement and finding target objects in complex scenes.

Children in the cohort with any ICD10 diagnosis (World Health Organization International Classification of Disease 10 [42]) that might affect development had already been identified by the ALSPAC study [43]. This had been achieved by requesting from the computer records of the 4 health trusts covering the study area details of children with any of a specified list of ICD10 diagnoses and with a date of birth that meant they were eligible for ALSPAC. This list included all children with a statement of Special Educational needs. These records were then matched with the ALSPAC dataset, and the hospital, outpatient and community notes for all identified children were reviewed by an experienced abstractor, to confirm the ICD10 diagnoses and add them to the ALSPAC dataset. The children's IQ was tested at the age of 8 in an ALSPAC research clinic using a shortened (alternate question) version of the Wechsler Intelligence Scales for Children (WISC III, UK version)[44], [45]. Visual abilities were examined by orthoptists in an ALSPAC clinic when the children were aged 7: observations included monocular visual acuity (with habitual correction +/− a pinhole as well), presence/type of strabismus and level of stereoacuity (depth perception). We used the prospectively-collected ALSPAC questionnaire data regarding the children's early medical history (birthweight, gestation at birth, whether admitted to intensive care or special care within first month)[23] and socioeconomic background to adjust for these potential predictors of school performance.

### Analysis

The predictor of interest (the child's VP abilities) was expressed as a raw score obtained by summing for each child their mother's responses to all the questions, and also by using principal components analysis (PCA) to condense the individual responses into 3 underlying “factors” or themes. These explained over half the variance in the responses to the VP questions. We used the VP skills as continuous variables (raw scores, factors 1–3) and we categorized the scores into quartiles to look for differences in effect across the range of VP abilities.

We used generalised linear models to examine unadjusted and adjusted associations between each indicator of VP abilities and the educational results. We expressed the results as parameter estimates (95% confidence limits) to facilitate comparisons between models. The adjusted models also included data on age at Key Stage (KS) testing in months; gender; highest level of maternal education (CSE or <11 years, vocational qualification only, O-level or 11 yrs, A –level or 13 years, degree); family social class (6 graded categories with the top 3 non-manual and the bottom 3 manual employment, highest of mother and her partner); any ICD10 diagnosis affecting development (yes/no); vision problems at 7 (any or none of strabismus, corrected acuity worse than 6/12 in best eye, stereopsis in lowest 20% of sample); born before 37 weeks (yes/no), birthweight lower than 2SD below the sample mean (yes/no); and total IQ at age 8. Additional models also adjusted for the child's performance in previous school tests on the same subject (KS2 reading and mathematics).

One problem that occurs when analyzing large datasets like the one used here is that of missing data. It is now recommended that investigators do not restrict all analyses to individuals with complete datasets, but that they also use methods to try to estimate or “impute” the missing values, based on relevant other information that is available for each participant with missing data [46]. We used multiple imputation by chained equation (MICE) to impute missing data. The imputation models included KS3 reading and maths scores, VP scores and predictors of “missingness”. We generated 25 datasets and undertook 10 switching procedures. The variables used to impute were all outcomes, the predictors used in the adjusted analyses and the ALSPAC “family adversity index”, a derived variable that summarises several variables that indicate family social or economic hardship (data not shown). We repeated the adjusted models using the imputed data to compare with the complete case analyses.
